# An insight into the electro-chemical properties of halogen (F, Cl and Br) doped BP and BN nanocages as anodes in metal-ion batteries

**DOI:** 10.1038/s41598-020-76749-0

**Published:** 2020-11-17

**Authors:** Maryam Abedi, Mohammad Eslami, Mahdi Ghadiri, Samira Mohammadinia

**Affiliations:** 1 Department of Chemical Engineering, Faculty of Imam Mohammad Bagher, Sari Branch, Technical and Vocational University (TVU), Mazandaran, Iran; 2 Department of Electrical and Computer Engineering, Chabahar Branch, Islamic Azad University, Chabahar, Iran; 3 Institute of Research and Development, Duy Tan University, Da Nang, 550000 Vietnam; 4 The Faculty of Environment and Chemical Engineering, Duy Tan University, Da Nang, 550000 Vietnam; 5 Department of Chemical Engineering, Islamic Azad University, Mahshahr Branch, Mahshahr, Iran

**Keywords:** Chemistry, Energy science and technology, Engineering, Materials science, Mathematics and computing, Physics

## Abstract

Here, electro-chemical properties of BN and BP nanocages as anodes in metal-ion batteries are examined. The effect of halogens adoption of BN and BP-NCs on electro-chemical properties of M-IBs are investigated. Results showed that the BP nanocages as anode electrode in M-IBs has higher efficiency than BN nanocages and the K-IB has higher cell voltage than N-IBs. Results indicated that the halogens adoption of BN and BP-NCs are improved the cell voltage of M-IBs. Results proved that the F-doped M-IBs have higher cell voltage than M-IBs. Finally, F-B_17_P_18_ as anodes in K-IB is proposed as suitable electrodes.

## Introduction

In previous studies, the chemical and physical properties of boron nitride nanocages (BN-NC) and boron phosphide nanocages (BP-NC) have been investigated^1–3^. The results of previous studies confirmed that nanocages have acceptable properties as anodes and cathode materials in batteries due to low band gap energies and high potential to transfer the electrons and ions^4–7^.

Results of previous studies indicated that the formation heat and formation heat of per atom of B_18_N_18_, B_24_N_24_ and B_36_N_36_ are decreased when the number of atoms are increased. Results of previous studies confirmed that the formation heat and formation heat of per atom of B_18_P_18_ is higher than those of B_24_P_24_ and B_36_P_36_, significantly^8–11^. Results of previous studies showed that the formation heat of B_18_N_18_, B_24_N_24_ and B_36_N_36_ are − 1275, − 1197 and – 1034 kcal/mol and the formation heat of per atom of B_18_N_18_, B_24_N_24_ and B_36_N_36_ are − 15, − 13 and – 12 kcal/mol^8–11^.

Results of previous studies indicated that the halogen (F, Cl and Br) adoption of nanocages are decreased the band gap energies of nanocages and halogen (F, Cl and Br) adoption are improved the electro-chemical properties (cell voltage) of nanocages as anodes and cathode materials in batteries^12–15^. The potential of graphite, nanotubes and nanocages as anodes of metal-ion batteries (M-IBs) have been studied and results showed that nanocages have higher potential rather graphite and nanotubes^16–18^.

In previous studies, the electro-chemical properties (cell voltage) of B_12_N_12_ as anodes in L-IBs and Na-IBs are examined and results confirmed that the F and Br are improved the properties of L-IBs and Na-IBs^19–21^. In previous studies, the metal adsorption on BN-NCs are investigated and results indicated that the lithium and potassium atoms are increased the properties of NC in M-IBs. The electronic properties of B_16_N_16_, B_16_P_16_ and B_7_C_24_P are examined and and results indicated that the electro-chemical properties (cell voltage) of BN nanocages are improved by increasing the size of rigs^22,23^.

Razavi et al.^24^ studied the roles of halogen on potential of B_18_N_18_ and B_18_P_18_ in MIBs by theoretical methods. They demonstrated that storage capacities of B_18_N_18_ and B_18_P_18_ in L-IBs are 893 and 795 mAh/g and they showed that V_cell_ of F-doped B_18_N_18_ and B_18_P_18_ are higher than Br-doped MIBs by theoretical calculation.

Tahvili et al.^25^ studied the potential of various nanocages as anodes in MIBs by theoretical methods. They indicated that Al_22_P_22_ is the suitable candidate as anode in MIBs and they showed that adsorbed halogen nanocages have higher V_cell_ than nanocages in MIBs. They proposed the F-Al_21_P_22_ nanocage as suitable material in anodes of MIBs by theoretical calculation.

In this study, electro-chemical properties of BN and BP-NCs as anodes in L-IBs, N-IBs and K-IBs are examined. The effects of F, Cl and Br doping of BN and BP-NCs on their electro-chemical properties (cell voltage) as anode electrodes in M-IBs are examined. The main goals of this study are to (1) find the cell voltages of LIBs made of BP and BN nanocages as anodes; (2) compare the cell voltages of LIBs and NIBs made of BP and BN nanocages as anodes; (3) find the effects of halogen adoption on cell voltages of LIBs made of BP and BN nanocages as anodes; (4) propose the metal-ion batteries with high cell voltage values.

## Computational details

The geometries of B_18_N_18_, B_18_P_18_, X-B_18_N_18_ and X-B_18_P_18_ (X = F Cl, Br) were optimized through *DFT* method*,* M06-2X and HSE06 functional and 6-31G (d, p) basis set in *GAMESS*^26–30^. The DFT is described energies of inorganic–organic nanocages with acceptable accuracy and M06-2X and HSE06 functional have high accurate to estimate the energies of nano-structures^31–35^. The DFT/M06-2X is predicted the energies and frequencies of nanocages with suitable accuracy and the M06 is the best functional to estimate the vibrational frequencies of nanocages^36–40^.

The adsorption of halogens (X= F Cl, Br) on nanocages and adsorption of M and M^+^ on nanocages are studied. The structures of X-nanocages and M-nanocages are optimized by *DFT* method*,* M06-2X and HSE06 functional and 6-31G (d, p) basis set. The vibrational frequencies of studied complexes are investigated by *DFT* method*,* M06-2X and HSE06 functional and 6-311+G (2d, 2p) basis set^41–43^.

The Gibbs free energy of structures are calculated as follow: *G* = *E*_0_ + *ZPE* + ∆*H*_trans_ + ∆*H*_rot_ + ∆*H*_vib_ + *RT-TS.* The Gibbs free energy of adsorption of halogens on nanocages are calculated as follow: *G*_ad_ = *G *(X-nanocage) − *G *(nanocage) − 0.5 *G *(X_2_)*.* The Gibbs free energy of adsorption of M and M^+^ on nanocages are calculated as follow: *G*_ad_ = *G *(M-nanocage) − *G *(nanocage) − *G* (M)^44–47^*.*

The reactions in anode and cathode of metal ion batteries are: Anode: M-nanocage ↔ M^+^-nanocage + e^*−*^ and Cathode: M^+^ + e^−^ ↔ M. The final reaction in metal-ion battery is: M^+^ + M-nanocage ↔ M^+^-nanocage + M. The cell voltage (*V*_cell_) is calculated by via Nernst equation: *V*_cell_ = − Δ*G*_cell_/*zF.* The *F* is the Faraday constant, *z* is the charge of M^+^ and Δ*G*_cell_ is Δ*E*_cell_ + *P*∆*V* − *T*∆*S*^48–51^*.*

## Results and discussion

### BN and BP as anodes in M-IBs

**Figure 1:**
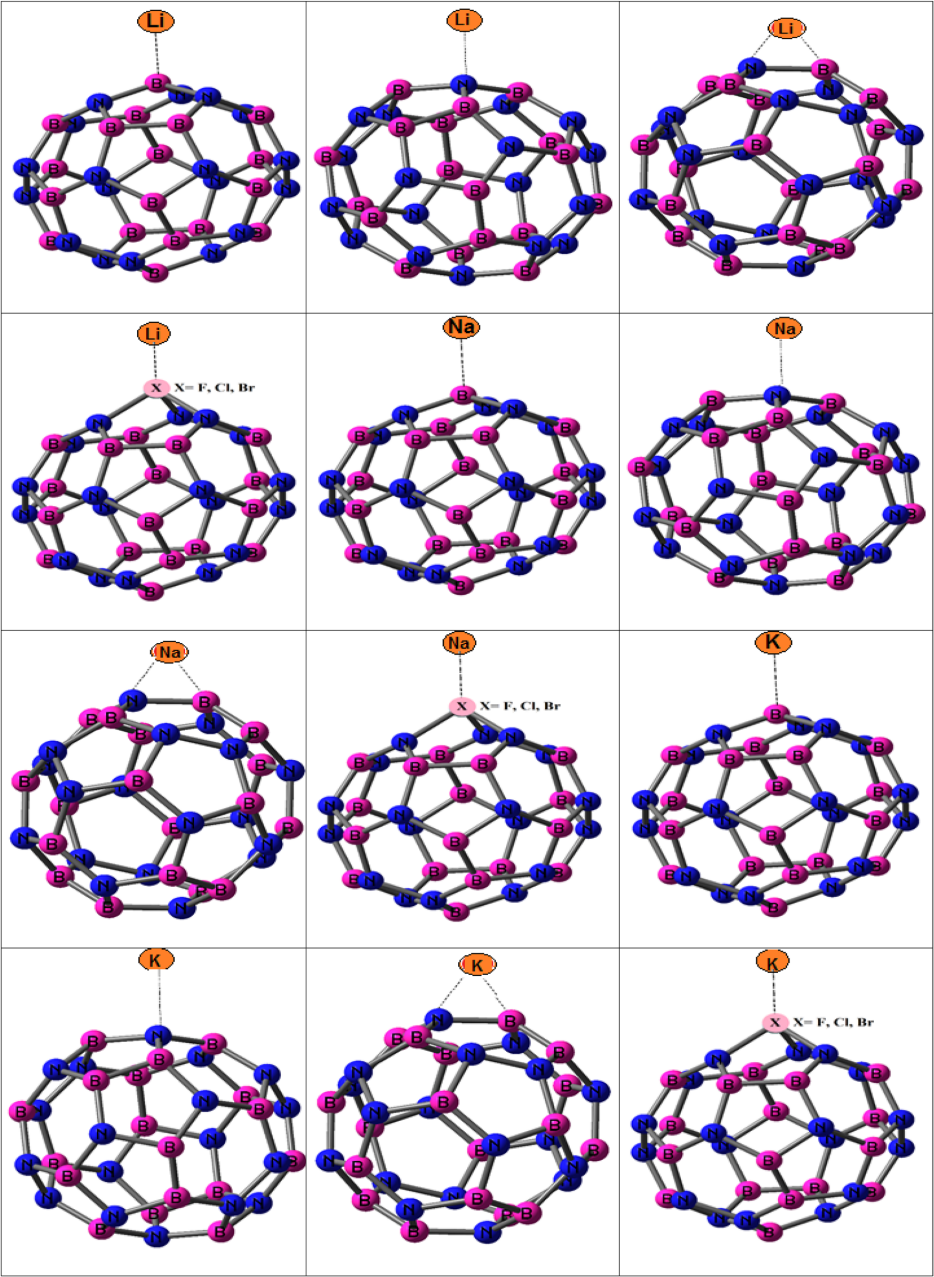

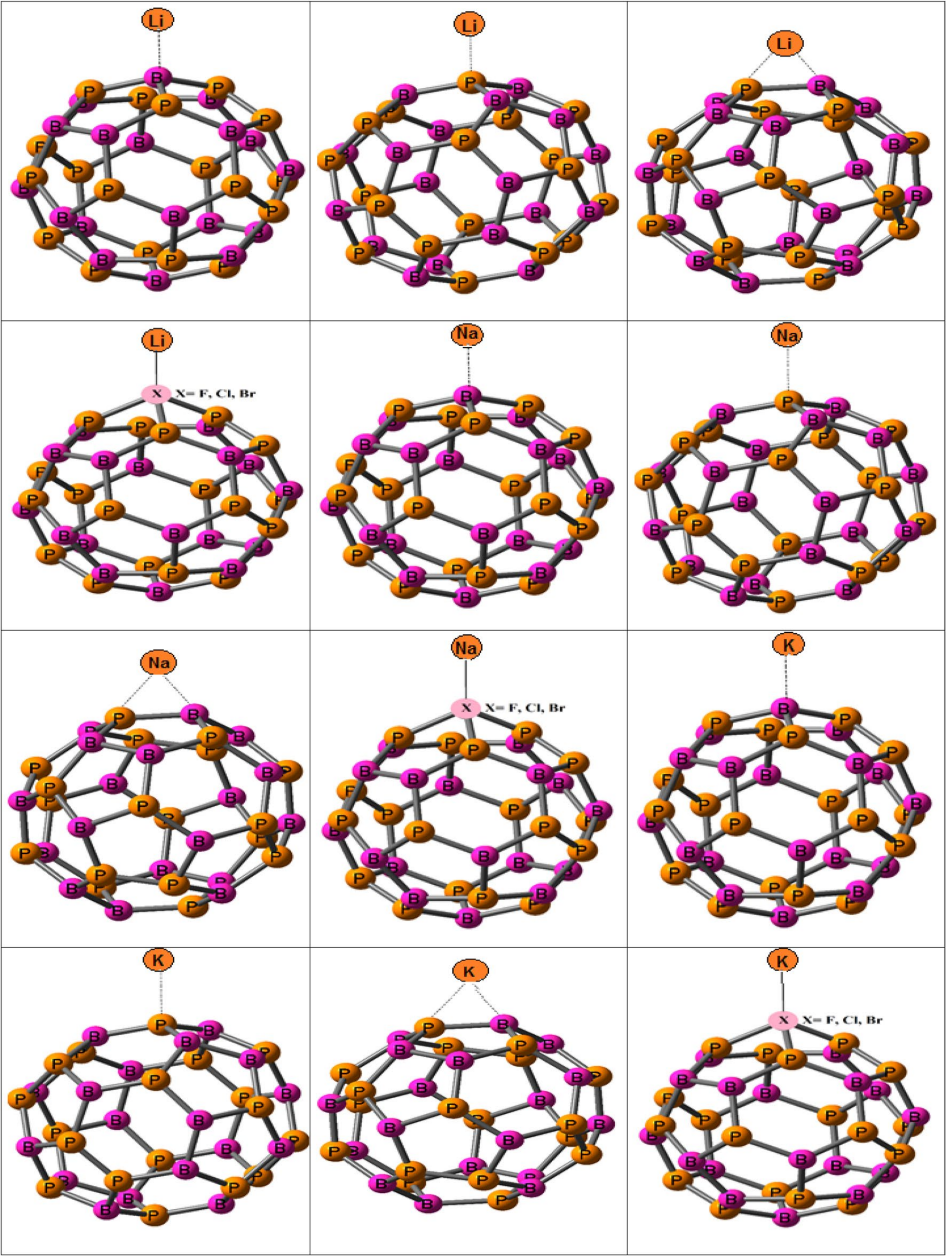
Structures of B_18_N_18_, B_18_P_18_, X-B_17_N_18_ and X-B_17_P_18_ with metal atoms and their bond lengths (Å) and calculated *G*_ad_ of adsorption of halogens on studied nanocages.

In this section, the structures of nanocages and complexes with halogens and metals are showed in Fig. 1. The electro-chemical properties of BN and BP-NCs as anodes in M-IBs are investigated. The structures and bond lengths (in Å) of BN and BP-NCs with metal are showed in Fig. 1.

The calculated *G*_ad_ of M/M^+^ and BN and BP-NCs by M06-2X and HSE06 functional are summarized in Supplementary Table 1S in supplementary data. The *G*_ad_ values are negative and adsorption of metal on BN and BP-NCs are possible. The |*G*_ad_| of K-B_18_N_18_ is higher than |*G*_ad_| of Li-B_18_N_18_ and Na-B_18_N_18_. The |*G*_ad_| of adsorption of metal on B_18_P_18_ are higher than |*G*_ad_| on B_18_N_18_. The |*G*_ad_| of studied M and M^+^ on BN and BP-NCs have same trends. The calculated *V*_cell_ of metal with BN and BP-NCs by M06-2X and HSE06 functional are reported in Table 1.

**Table 1:** The calculated *V*_cell_ of BN and BP nanostructures.

Position	Complex	*V*_cell_ by M06-2X	*V*_cell_ by HSE06	Position	Structure	*V*_cell_ by M06-2X	*V*_cell_ by HSE06
B site	K-B_18_N_18_	1.39	1.43	B site	K-B_18_P_18_	1.60	1.64
B site	Na-B_18_N_18_	1.24	1.26	B site	Na-B_18_P_18_	1.43	1.45
B site	Li-B_18_N_18_	1.11	1.14	B site	Li-B_18_P_18_	1.28	1.32
N site	K-B_18_N_18_	1.41	1.44	P site	K-B_18_P_18_	1.63	1.67
N site	Na-B_18_N_18_	1.27	1.30	P site	Na-B_18_P_18_	1.45	1.48
N site	Li-B_18_N_18_	1.13	1.17	P site	Li-B_18_P_18_	1.31	1.35
Bridge B-N	K-B_18_N_18_	1.37	1.41	Bridge B-P	K-B_18_P_18_	1.57	1.62
Bridge B-N	Na-B_18_N_18_	1.22	1.26	Bridge B-P	Na-B_18_P_18_	1.41	1.45
Bridge B-N	Li-B_18_N_18_	1.08	1.11	Bridge B-P	Li-B_18_P_18_	1.25	1.29
K-F-B_17_N_18_		3.12	3.22	K-F-B_17_P_18_		3.59	3.70
Na-F-B_17_N_18_		2.78	2.87	Na-F-B_17_P_18_		3.20	3.30
Li-F-B_17_N_18_		2.49	2.57	Li-F-B_17_P_18_		2.86	2.96
K-Cl-B_17_N_18_		2.95	3.04	K-Cl-B_17_P_18_		3.39	3.50
Na-Cl-B_17_N_18_		2.64	2.70	Na-Cl-B_17_P_18_		3.03	3.11
Li-Cl-B_17_N_18_		2.35	2.45	Li-Cl-B_17_P_18_		2.71	2.82
K-Br-B_17_N_18_		2.78	2.89	K-Br-B_17_P_18_		3.20	3.33
Na-Br-B_17_N_18_		2.49	2.58	Na-Br-B_17_P_18_		2.86	2.96
Li-Br-B_17_N_18_		2.22	2.29	Li-Br-B_17_P_18_		2.55	2.64

The *V*_cell_ of K-B_18_N_18_ is higher than Li-B_18_N_18_ and Na-B_18_N_18_ and *V*_cell_ of K-B_18_P_18_ is higher than Li-B_18_P_18_ and Na-B_18_P_18_. The *V*_cell_ of Li, Na and K on B_18_P_18_ are higher than B_18_N_18_. Here, interactions of atoms on B atom in BN and BP-NCs are examined. The structures of M atoms with BN and BP-NCs via N and P sites are showed in Fig. 1. Calculated *V*_cell_ of BN and BP-NCs with M atoms via N and P sites by M06-2X and HSE06 functional are reported in Table 1.

The |*G*_ad_| of M atoms with BN and BP-NCs via N and P sites are higher than |*G*_ad_| of B site ca 0.13 kcal/mol. The |*G*_ad_| of M^+^ with BN and BP-NCs via N and P sites are higher than |*G*_ad_| of B site ca 0.75 kcal/mol.

The |*G*_ad_| of M atoms on BN and BP-NCs via N and P sites are higher than |*G*_ad_| of bridge B-N and B-P ca 0.28 kcal/mol. The |*G*_ad_| of M^+^ with BN and BP-NCs via N and P sites are higher than |*G*_ad_| of bridge B-N and B-P site ca 1.45 kcal/mol. Results showed that the trends of calculated *G*_ad_ by M06-2X and HSE06 functional are same for studied nanocages.

The *V*_cell_ of M atoms with BN and BP-NCs via N and P sites are higher than *V*_cell_ of B site and bridge site ca 0.03 and 0.05 V. Finally, M atoms on BN and BP-NCs via N and P sites are more stable than B site and bridge site.

The calculated orbital energies of metal with BN and BP-NCs by M06-2X and HSE06 functional are described in Supplementary Table 2S in supplementary data. The |*E*_HOMO_| of metal with B_18_P_18_ are smaller than |*E*_HOMO_| of metal with B_18_N_18_. The *E*_HLG_ of K-B_18_N_18_ is smaller than *E*_HLG_ of Li-B_18_N_18_ and Na-B_18_N_18_.

Results show that the trends of calculated *E*_HOMO_, *E*_LUMO_ and *E*_HLG_ by M06-2X and HSE06 functional are same for studied nanocages. The B_18_P_18_ in M-IBs has higher efficiency than B_18_N_18_ and KIB has higher *V*_cell_ than NIB and KIB.

### F, Cl and Br doped BN and BP nano-structures as anodes in MIBs

The calculated G_ad_ of F-, Cl- and Br-doped BN-NCs and BP-NCs by M06-2X and HSE06 functional are summarized in Supplementary Table 1S in supplementary data. The G_ad_ values are negative and halogens adoption of BN and BP-NCs are possible, from thermodynamic view point. The |G_ad_| of X-B_17_N_18_ and X-B_17_P_18_ are higher than X-B_18_N_17_ and X-B_18_P_17_ ca 2.07 and 2.05 kcal/mol. The B atoms of BN and BP-NCs are suitable to replace with halogen atoms.

The |G_ad_| of F-B_17_N_18_ is higher than Cl-B_17_N_18_ and Br-B_17_N_18_. The |G_ad_| of F-B_17_P_18_ is higher than Cl-B_17_P_18_ and Br-B_17_P_18_. The |G_ad_| of doping of B_18_P_18_ with halogens are higher than B_18_N_18_. Doping of BN and BP-NCs with F is possible process from thermodynamic view point and F-B_17_N_18_ and F-B_17_P_18_ are acceptable candidates in M-IBs.

The structures and bond lengths of halogen doped nanocages with metals are showed in Fig. 1. The G_ad_ of M and M^+^ with halogen doped nanocages are presented in Supplementary Table 1S in supplementary data. The G_ad_ values are negative and adsorption of meals on halogen doped nanocages are possible.

The G_ad_ of K-halogen-B_17_P_18_ are higher than Na-halogen-B_17_P_18_ and Li-halogen-B_17_P_18_. The |G_ad_| of adsorption of metals on halogen-B_17_P_18_ are higher than halogen-B_17_N_18_. The |G_ad_| of F-NCs are higher than Cl-NCs and Br-NCs. The orbital energies of metals halogen doped nanocages are reported in Supplementary Table 2S in supplementary data. The |E_HOMO_| of metals with halogen-B_17_P_18_ are smaller than halogen-B_17_N_18,_ significantly.

The calculated V_cell_ of metals with halogen doped nanocages by M06-2X and HSE06 functional are reported in Table 1. The V_cell_ of K-B_17_N_18_ are higher than Li-B_17_N_18_ and Na-B_17_N_18_. The V_cell_ of K-B_17_P_18_ are higher than Li-B_17_P_18_ and Na-B_17_P_18_. The V_cell_ of metals with halogen-B_17_P_18_ are higher than metals with halogen-B_17_N_18_. Results show that the trends of calculated V_cell_ by M06-2X and HSE06 functional are same for studied nanocages.

In present paper, F doping of BN and BP-NCs is increased the *V*_cell_ of them in M-IBs. The *V*_cell_ of F-B_17_N_18_ and F-B_17_P_18_ in N-IB are higher than *V*_cell_ of F-B_17_N_18_ and F-B_17_P_18_ in L-IB. The halogens doping of NCs are increased the *V*_cell_ of M-IBs. The K-ion batteries have higher *V*_cell_ than M-IBs and the K-F-B_17_P_18_ in M-IBs has the highest *V*_cell_.

## Conclusion

In this study, the electro-chemical properties of BN and BP-NCs as anodes in M-IBs are examined. The roles of halogens adoption on electro-chemical properties of BN-NCs and BP-NCs as anodes of metal-IB are investigated. The obtained results of this study are: (1) the B_18_P_18_ as anode electrode of M-IBs has higher efficiency than B_18_N_18_; (2) the KIB has higher *V*_cell_ than NIB and KIB; (3) halogens are increased *V*_cell_ in M-IBs; (4) the F doped NCs have higher *V*_cell_ than Cl and Br doped NCs in M-IBs; (5) F-B_17_P_18_ has the highest *V*_cell_ as anode electrodes in K-IB and F-B_17_P_18_ is proposed as novel anodes in M-IBs.

## Supplementary information

Supplementary Tables.

## Data Availability

The calculated G_ad_ of nano-structures by M06-2X and HSE06 functional are presented in Table 1S and calculated energies of orbitals and *q* of nano-structures by M06-2X and HSE06 functional are presented in Table 2S.
