# Supplementary material for: An insight into the electro-chemical properties of halogen (F, Cl and Br) doped BP and BN nanocages as anodes in metal-ion batteries
Source: Sci Rep. 2020 Nov 17;10:19948. doi: 10.1038/s41598-020-76749-0 (PMC7672099; doi:10.1038/s41598-020-76749-0)
Supplement: Supplementary file 1 — Supplementary Tables. [file 41598_2020_76749_MOESM1_ESM.docx]

**An insight into the electro-chemical properties of halogen (F, Cl and Br) doped BP and BN nanocages as anodes in metal-ion batteries**

*Maryam Abedi^1^, Mohammad Eslami^2^, Mahdi Ghadiri^3,4,*^, Samira Mohammadinia^5,*^*

*^1^Department of Chemical Engineering, Faculty of Imam Mohammad Bagher, sari branch, Technical and Vocational University (TVU), Mazandaran, Iran.*

*^2^ Department of Electrical and Computer Engineering, Chabahar branch, Islamic Azad University, Chabahar, Iran*

*^3^ Institute of Research and Development, Duy Tan University, Da Nang 550000, Vietnam*

*^4^The Faculty of Environment and Chemical Engineering, Duy Tan University, Da Nang 550000, Vietnam*

*^5^ Department of chemical engineering, Islamic Azad University, Mahshahr branch, Mahshahr, Iran*

**Corresponding authors: mahdighadiri@duytan.edu.vn (M. Ghadiri),* [*samiramohammadinia@gmail.com*](mailto:samiramohammadinia@gmail.com) *(S. Mohammadinia)*

**Table 1S.** *G*_ad_ of complexes of nanocages with metals and metal ions.

| **Position** | **Complex** | ***G*_ad_ by M06-2X** | ***G*_ad_ by HSE06** | **Position** | **Structure** | ***G*_ad_ by M06-2X** | ***G*_ad_ by HSE06** |
| --- | --- | --- | --- | --- | --- | --- | --- |
| **B site** | **K-B_18_N_18_** | -7.69 | -8.54 | **B site** | **K-B_18_P_18_** | -8.85 | -9.82 |
| **B site** | **Na-B_18_N_18_** | -6.87 | -7.69 | **B site** | **Na-B_18_P_18_** | -7.9 | -8.85 |
| **B site** | **Li-B_18_N_18_** | -6.13 | -6.93 | **B site** | **Li-B_18_P_18_** | -7.05 | -7.97 |
| **N site** | **K-B_18_N_18_** | -7.82 | -8.76 | **P site** | **K-B_18_P_18_** | -8.99 | -10.07 |
| **N site** | **Na-B_18_N_18_** | -7.01 | -7.78 | **P site** | **Na-B_18_P_18_** | -8.03 | -8.91 |
| **N site** | **Li-B_18_N_18_** | -6.25 | -7.06 | **P site** | **Li-B_18_P_18_** | -7.19 | -8.12 |
| **Bridge B-N** | **K-B_18_N_18_** | -7.54 | -8.44 | **Bridge B-P** | **K-B_18_P_18_** | -8.71 | -9.76 |
| **Bridge B-N** | **Na-B_18_N_18_** | -6.71 | -7.45 | **Bridge B-P** | **Na-B_18_P_18_** | -7.72 | -8.57 |
| **Bridge B-N** | **Li-B_18_N_18_** | -5.99 | -6.77 | **Bridge B-P** | **Li-B_18_P_18_** | -6.89 | -7.79 |
| **B type** | **K^+^-B_18_N_18_** | -39.75 | -41.42 | **B type** | **K^+^-B_18_P_18_** | -45.72 | -47.64 |
| **B type** | **Na^+^-B_18_N_18_** | -35.5 | -36.81 | **B type** | **Na^+^-B_18_P_18_** | -40.82 | -42.33 |
| **B type** | **Li^+^-B_18_N_18_** | -31.69 | -33.31 | **B type** | **Li^+^-B_18_P_18_** | -36.44 | -38.30 |
| **N type** | **K^+^-B_18_N_18_** | -40.34 | -41.91 | **P type** | **K^+^-B_18_P_18_** | -46.69 | -48.51 |
| **N type** | **Na^+^-B_18_N_18_** | -36.33 | -37.78 | **P type** | **Na^+^-B_18_P_18_** | -41.44 | -43.10 |
| **N type** | **Li^+^-B_18_N_18_** | -32.38 | -34.03 | **P type** | **Li^+^-B_18_P_18_** | -37.29 | -39.19 |
| **Bridge B-N** | **K^+^-B_18_N_18_** | -39.02 | -40.89 | **Bridge B-P** | **K^+^-B_18_P_18_** | -45.01 | -47.17 |
| **Bridge B-N** | **Na^+^-B_18_N_18_** | -34.81 | -36.41 | **Bridge B-P** | **Na^+^-B_18_P_18_** | -40.15 | -42.00 |
| **Bridge B-N** | **Li^+^-B_18_N_18_** | -30.96 | -32.45 | **Bridge B-P** | **Li^+^-B_18_P_18_** | -35.75 | -37.47 |
| **K-F-B_17_N_18_** | | -17.24 | -19.31 | **K-F-B_17_P_18_** | | -19.82 | -22.20 |
| **Na-F-B_17_N_18_** | | -15.39 | -17.39 | **Na-F-B_17_P_18_** | | -17.70 | -20.00 |
| **Li-F-B_17_N_18_** | | -13.74 | -15.25 | **Li-F-B_17_P_18_** | | -15.80 | -17.54 |
| **K-Cl-B_17_N_18_** | | -16.31 | -18.10 | **K-Cl-B_17_P_18_** | | -18.76 | -20.82 |
| **Na-Cl-B_17_N_18_** | | -14.57 | -16.46 | **Na-Cl-B_17_P_18_** | | -16.75 | -18.93 |
| **Li-Cl-B_17_N_18_** | | -13.01 | -14.57 | **Li-Cl-B_17_P_18_** | | -14.96 | -16.76 |
| **K-Br-B_17_N_18_** | | -15.39 | -17.39 | **K-Br-B_17_P_18_** | | -17.70 | -20.00 |
| **Na-Br-B_17_N_18_** | | -13.74 | -15.25 | **Na-Br-B_17_P_18_** | | -15.80 | -17.54 |
| **Li-Br-B_17_N_18_** | | -12.27 | -13.74 | **Li-Br-B_17_P_18_** | | -14.11 | -15.80 |
| **K^+^-F-B_17_N_18_** | | -89.06 | -93.60 | **K^+^-F-B_17_P_18_** | | -102.41 | -107.63 |
| **Na^+^-F-B_17_N_18_** | | -79.51 | -83.64 | **Na^+^-F-B_17_P_18_** | | -91.44 | -96.19 |
| **Li^+^-F-B_17_N_18_** | | -70.99 | -74.54 | **Li^+^-F-B_17_P_18_** | | -81.64 | -85.72 |
| **K^+^-Cl-B_17_N_18_** | | -84.28 | -88.33 | **K^+^-Cl-B_17_P_18_** | | -96.93 | -101.58 |
| **Na^+^-Cl-B_17_N_18_** | | -75.26 | -78.80 | **Na^+^-Cl-B_17_P_18_** | | -86.54 | -90.61 |
| **Li^+^-Cl-B_17_N_18_** | | -67.20 | -71.03 | **Li^+^-Cl-B_17_P_18_** | | -77.28 | -81.68 |
| **K^+^-Br-B_17_N_18_** | | -79.51 | -84.12 | **K^+^-Br-B_17_P_18_** | | -91.44 | -96.74 |
| **Na^+^-Br-B_17_N_18_** | | -70.99 | -74.68 | **Na^+^-Br-B_17_P_18_** | | -81.64 | -85.89 |
| **Li^+^-Br-B_17_N_18_** | | -63.39 | -66.62 | **Li^+^-Br-B_17_P_18_** | | -72.90 | -76.62 |

**Table 2S.** Energies and *q* of metals with nano-structures.

| **Data by M06-2X** | | | | |
| --- | --- | --- | --- | --- |
| **Structures** | ***E*_HOMO_** | ***E*_LUMO_** | ***E*_HLG_** | ***q*** |
| **K-B_18_N_18_** | -4.22 | -1.17 | 3.05 | 0.53 |
| **Na-B_18_N_18_** | -4.31 | -1.07 | 3.24 | 0.46 |
| **Li-B_18_N_18_** | -4.42 | -0.95 | 3.47 | 0.42 |
| **K-F-B_17_N_18_** | -3.65 | -2.62 | 1.04 | 1.08 |
| **Na-F-B_17_N_18_** | -3.90 | -2.34 | 1.56 | 0.97 |
| **Li-F-B_17_N_18_** | -4.13 | -2.08 | 2.05 | 0.84 |
| **K-Cl-B_17_N_18_** | -3.77 | -2.47 | 1.30 | 1.01 |
| **Na-Cl-B_17_N_18_** | -4.01 | -2.21 | 1.80 | 0.94 |
| **Li-Cl-B_17_N_18_** | -4.23 | -1.97 | 2.25 | 0.87 |
| **K-Br-B_17_N_18_** | -3.90 | -2.34 | 1.56 | 0.97 |
| **Na-Br-B_17_N_18_** | -4.13 | -2.08 | 2.05 | 0.89 |
| **Li-Br-B_17_N_18_** | -4.33 | -1.86 | 2.47 | 0.76 |
| **K-B_18_P_18_** | -3.57 | -1.31 | 2.66 | 0.62 |
| **Na-B_18_P_18_** | -4.08 | -1.17 | 2.89 | 0.56 |
| **Li-B_18_P_18_** | -4.12 | -1.09 | 3.03 | 0.50 |
| **K-F-B_17_P_18_** | -3.55 | -2.83 | 0.71 | 1.25 |
| **Na-F-B_17_P_18_** | -3.66 | -2.70 | 0.96 | 1.17 |
| **Li-F-B_17_P_18_** | -3.87 | -2.40 | 1.47 | 1.08 |
| **K-Cl-B_17_P_18_** | -3.63 | -2.70 | 0.93 | 1.19 |
| **Na-Cl-B_17_P_18_** | -3.76 | -2.55 | 1.21 | 1.08 |
| **Li-Cl-B_17_P_18_** | -3.95 | -2.28 | 1.67 | 0.92 |
| **K-Br-B_17_P_18_** | -3.71 | -2.60 | 1.11 | 1.13 |
| **Na-Br-B_17_P_18_** | -3.87 | -2.40 | 1.47 | 1.01 |
| **Li-Br-B_17_P_18_** | -4.04 | -2.14 | 1.90 | 0.89 |
| **Data by HSE06** | | | | |
| **Structures** | ***E*_HOMO_** | ***E*_LUMO_** | ***E*_HLG_** | ***q*** |
| **K-B_18_N_18_** | -4.72 | -1.32 | 3.40 | 0.60 |
| **Na-B_18_N_18_** | -4.85 | -1.20 | 3.65 | 0.51 |
| **Li-B_18_N_18_** | -4.96 | -1.06 | 3.90 | 0.48 |
| **K-F-B_17_N_18_** | -4.12 | -2.93 | 1.19 | 1.22 |
| **Na-F-B_17_N_18_** | -4.35 | -2.62 | 1.74 | 1.08 |
| **Li-F-B_17_N_18_** | -4.62 | -2.33 | 2.29 | 0.95 |
| **K-Cl-B_17_N_18_** | -4.22 | -2.78 | 1.43 | 1.14 |
| **Na-Cl-B_17_N_18_** | -4.49 | -2.49 | 2.00 | 1.05 |
| **Li-Cl-B_17_N_18_** | -4.76 | -2.22 | 2.54 | 0.99 |
| **K-Br-B_17_N_18_** | -4.38 | -2.63 | 1.75 | 1.09 |
| **Na-Br-B_17_N_18_** | -4.62 | -2.33 | 2.29 | 0.99 |
| **Li-Br-B_17_N_18_** | -4.87 | -2.08 | 2.79 | 0.86 |
| **K-B_18_P_18_** | -4.01 | -1.46 | 2.54 | 0.69 |
| **Na-B_18_P_18_** | -4.60 | -1.31 | 3.29 | 0.64 |
| **Li-B_18_P_18_** | -4.60 | -1.22 | 3.38 | 0.56 |
| **K-F-B_17_P_18_** | -3.97 | -3.16 | 0.81 | 1.39 |
| **Na-F-B_17_P_18_** | -4.10 | -3.03 | 1.06 | 1.33 |
| **Li-F-B_17_P_18_** | -4.35 | -2.69 | 1.67 | 1.22 |
| **K-Cl-B_17_P_18_** | -4.07 | -3.02 | 1.06 | 1.34 |
| **Na-Cl-B_17_P_18_** | -4.21 | -2.85 | 1.36 | 1.20 |
| **Li-Cl-B_17_P_18_** | -4.44 | -2.56 | 1.89 | 1.04 |
| **K-Br-B_17_P_18_** | -4.16 | -2.92 | 1.24 | 1.27 |
| **Na-Br-B_17_P_18_** | -4.36 | -2.69 | 1.68 | 1.13 |
| **Li-Br-B_17_P_18_** | -4.51 | -2.39 | 2.12 | 1.01 |
